# Polyester Nanocapsules for Intravenous Delivery of Artemether: Formulation Development, Antimalarial Efficacy, and Cardioprotective Effects In Vivo

**DOI:** 10.3390/polym14245503

**Published:** 2022-12-15

**Authors:** Alessandra Teixeira Vidal-Diniz, Homero Nogueira Guimarães, Giani Martins Garcia, Érika Martins Braga, Sylvain Richard, Andrea Grabe-Guimarães, Vanessa Carla Furtado Mosqueira

**Affiliations:** 1 School of Pharmacy, Universidade Federal de Ouro Preto (UFOP), Campus Universitário Morro do Cruzeiro, Ouro Preto 35400-000, MG, Brazil; 2 Department of Electrical Engineering, Federal University of Minas Gerais, Belo Horizonte 31270-901, MG, Brazil; 3 Department of Parasitology, Institute of Biological Sciences, Universidade Federal de Minas Gerais (UFMG), Belo Horizonte 31270-901, MG, Brazil; 4 CNRS, INSERM, Université de Montpellier, 34295 Montpellier, France; 5 PhyMedExp, CHU Arnaud de Villeneuve 371, Avenue du Doyen Gaston Giraud, CEDEX 05, 34295 Montpellier, France

**Keywords:** nanocapsules, QT interval, cardiotoxicity, artemether, malaria, self-assembled polymers, polylactide, drug delivery

## Abstract

Artemether (ATM) is an effective antimalarial drug that also has a short half-life in the blood. Furthermore, ATM is also cardiotoxic and is associated with pro-arrhythmogenic risks. We aimed to develop a delivery system enabling the prolonged release of ATM into the blood coupled with reduced cardiotoxicity. To achieve this, we prepared polymeric nanocapsules (NCs) from different biodegradable polyesters, namely poly(*D,L*-lactide) (PLA), poly-ε-caprolactone (PCL), and surface-modified NCs, using a monomethoxi-polyethylene glycol-*block*-poly(*D,L*-lactide) (PEG_5kDa_-PLA_45kDa_) polymer. Using this approach, we were able to encapsulate high yields of ATM (>85%, 0–4 mg/mL) within the oily core of the NCs. The PCL-NCs exhibited the highest percentage of ATM loading as well as a slow release rate. Atomic force microscopy showed nanometric and spherical particles with a narrow size dispersion. We used the PCL NCs loaded with ATM for biological evaluation following IV administration. As with free-ATM, the ATM-PCL-NCs formulation exhibited potent antimalarial efficacy using either the “Four-day test” protocol (ATM total at the end of the 4 daily doses: 40 and 80 mg/kg) in Swiss mice infected with *P. berghei* or a single low dose (20 mg/kg) of ATM in mice with higher parasitemia (15%). In healthy rats, IV administration of single doses of free-ATM (40 or 80 mg/kg) prolonged cardiac QT and QTc intervals and induced both bradycardia and hypotension. Repeated IV administration of free-ATM (four IV doses at 20 mg/kg every 12 h for 48 h) also prolonged the QT and QTc intervals but, paradoxically, induced tachycardia and hypertension. Remarkably, the incorporation of ATM in ATM-PCL-NCs reduced all adverse effects. In conclusion, the encapsulation of ATM in biodegradable polyester NCs reduces its cardiovascular toxicity without affecting its antimalarial efficacy.

## 1. Introduction

In 2020, nearly half of the world population was at risk of malaria with 241 million reported cases. Low immunity patients, such as infants, children under five years of age, pregnant women, and HIV/AIDS patients, are at the highest risk of developing severe malaria [1]. In the last 10 years, malaria has taken a higher toll on children from Africa, accounting for about 80% of all malaria deaths in this region [2]. Severe malaria is a complicated condition that requires rapid intravenous treatment with fast-acting antimalarial drugs in hospitals. In this serious condition, the majority of the patients are infected by *Plasmodium falciparum,* and the gold standard therapy recommended is based on artemisinin derivatives [1,2,3].

Modern strategies used to treat severe malaria include the development of new therapeutic agents and also optimization of old drugs using new formulations [4,5]. Artemisinin and its derivatives, such as artemether (ATM), are among the most potent antimalarial drugs and have fast action against intraerythrocytic forms of the parasite in the blood. Furthermore, ATM is active against species and strains of chloroquine-resistant *Plasmodium*. ATM is also effective against *Schistosoma* spp. infections and is currently under evaluation for cancer treatment repurposing [6,7]. However, the therapeutic use and safety of ATM need improvement. Limiting issues have been identified. They include short plasma half-life [8,9], low oral bioavailability (~30%), chemical degradation, and high risk of toxicity, especially in the cardiovascular system [10]. Additionally, the ATM dosage for intramuscular (IM) injection is associated with poor patient compliance and higher risks of neurotoxicity [10]. In this sense, a safe formulation of ATM administered intravenously (IV) is urgently required to treat severe malaria. ATM is a methyl ether derivative of artemisinin and a lipophilic drug with a log *P* of 3.48 [10,11]. Thus, it is a suitable candidate to be associated with biodegradable nanometric carriers containing lipid reservoirs, allowing prolonged delivery of ATM into the blood.

The strategy of encapsulating existing drugs within nanocarriers for the treatment of malaria is one of the most promising approaches currently available [4,12,13]. Polymeric nanometric devices have demonstrated an outstanding ability to reduce the toxicity of drugs [14,15,16,17] and to control their release into the blood [11,18,19,20]. They act by modifying drug biodistribution and release rates in the heart [18,19,21,22]. Different polyesters can generate nanocarriers able to load high levels of lipophilic molecules [14,20]. Biodegradable and biocompatible polyesters, such as polycaprolactone [14,16], polylactides [14], polylactide-*co*-glycolide [23], and amphiphilic diblock polymers (PEG-PLA) [19,24,25], efficiently produce oil-core nanocapsules (NCs), where drugs with high lipophilicity can be encapsulated. In these biocompatible nanosystems, the drugs remain protected from degradation and they are released over a longer period into the blood with reduced toxicity even after IV administration of high doses [14,15,18,19,26]. ATM has been associated with different types of nanocarriers [21,27,28,29]. Among them, NCs have demonstrated their potential to reduce the cardiotoxicity of the ATM in vitro and in vivo when administered orally [21,22]. Nanoencapsulation of another antimalarial drug, halofantrine, has also reduced its cardiotoxicity in vivo [14].

The artemisinin derivatives, including ATM, can cause a prolonged cardiac QT interval and lead to potentially fatal cardiac arrhythmias [30,31,32]. Although the mechanism of the QT prolongation may be secondary to central nervous system toxicity [31], it has been demonstrated that there are also direct acute myogenic effects. Indeed, ATM lengthens the action potential of ventricular cardiomyocytes, explaining the QTc prolongation, and disrupts Ca^2+^ handling. Both effects promote pro-arrhythmogenic risks [21]. Interestingly, encapsulation of ATM in NCs can prevent these adverse effects when administered orally [21,22].

The encapsulation of ATM in NCs could be useful to improve its efficacy and reduce its cardiovascular toxicity when administered IV during severe malaria treatment. To investigate this strategy, we developed polymeric NCs from three different biodegradable polyesters. Next, we validated their efficacy to treat experimental malaria in vivo in *Plasmodium berghei*-infected mice. Finally, we assessed the cardiovascular toxicity following IV administration of ATM, free or loaded, in optimized PCL NCs (ATM-PCL-NCs). 

## 2. Material and Methods

### 2.1. Drugs and REAGENTS

The National Malaria Control Program (Ministry of Health in Brazil) provided the ATM dissolved in oil solution for IM injection (PALUTHER^®^ 80 mg/mL). We purchased ATM (dihydroartemisinin methyl ether), poly-ε-caprolactone (PCL) polymer Mn 42,500 g/mol, Poly(*D,L*-lactic) acid (PLA) Mn 30,000 g/mol and Mw 60,000 g/mol, Poloxamer 188 (Pluronic F68), and HPLC grade acetone from Sigma-Aldrich (Sigma-Aldrich Co., St. Louis, MO, USA). We synthesized and characterized diblock PEG-PLA polymer (Mn 45,000 g/mol, Ð 1.18, copolymerized with PEG 5000 g/mol, Ð:1.03), with approximately 10% *w*/*w* PEG as described [33]. Cargill (Berlin, Germany) generously donated soy lecithin with approximately 75% of phosphatidylcholine. Sasol (Hamburg, Germany GmBH) provided medium-chain triglyceride (Miglyol^®^810N). Synth (Rio de Janeiro, Brazil) supplied polyethylene glycol (PEG 300). The solvents were all of the analytical grade or HPLC grade. We purified the water used throughout the experiments with the MilliQ^®^ system (Symplicity System 185, Millipore, Burlington, MA, USA).

### 2.2. Preparation of Nanocapsules and Intravenous Solution of Artemether

We prepared three NCs formulations (PCL, PLA, and PEG-PLA) by the polymer deposition method followed by solvent displacement [34]. We dissolved sixty milligrams of a polymer (PCL, PLA, or PEG-PLA) in 10 mL of an acetone solution containing 75 mg of Epikuron^®^170, and 250 μL of ATM oily solution (80 mg/mL in sesame oil for IM injection). We poured this organic solution into 20 mL of external aqueous phase under magnetic stirring using a syringe. The aqueous phase contained 75 mg of Poloxamer^®^188 for PCL and PLA NCs preparations and only water for PEG-PLA NCs preparation. After 10 min under agitation, we removed the solvents under reduced pressure in a rotary evaporator (Heildolph Rotary Evaporator Instruments, Schwabach, Germany) until a final 10 mL volume of NCs colloidal dispersion with 2 mg/mL of ATM. We prepared the unloaded NCs (blank-NCs) with the same method, replacing the ATM oily solution with 250 μL of Miglyol 810N oil. We obtained the ATM aqueous solution for IV injection by dissolving ATM powder in a dimethylacetamide/PEG 300/glucose 5 % (*w*/*v*) (2:4:96) solution. We protected free-ATM solution and ATM loaded in NCs from light exposure throughout the experiments. All the concentrations used for the different diluents were adequate for IV administration [35].

### 2.3. Nanocapsules Characterization

#### 2.3.1. Hydrodynamic Diameter and Zeta Potential Determination

We determined the mean hydrodynamic diameter and the polydispersity index (PI) for the size distribution of NCs at 25 °C, using dynamic light scattering (DLS) technique for size and electrophoretic laser doppler anemometry for zeta potential determinations on the same equipment, a Zetasizer NanoZS equipment (Malvern Instruments, Malvern, UK). We analyzed the samples for size measurements after appropriate dilution in ultra-pure Milli-Q water and for zeta potential in 1mM NaCl solution at a final conductivity close to 1.2 ± 0.2 mS/cm^2^. We expressed the reported values as the means ± standard deviations (SD) of at least three different batches of each NCs formulation. 

#### 2.3.2. Atomic Force Microscopy (AFM)

We performed a morphological analysis of the NCs by scanning probe microscopy in atomic force mode (AFM), using the equipment Multimode and Dimension 300, both monitored by NanoScope IIIa controller (Digital Instruments, Santa Barbara, Malvern, UK). We obtained the images in “tapping mode”, using silicon probes of 228 μm length, with a resonance frequency of 75–98 kHz, force constant of 29–61 N/m, and a nominal tip radius of curvature of 5 to 10 nm. We obtained the images by depositing approximately 5 μL of NCs samples on a freshly cleaved mica surface. After deposition on mica, we dried the samples using argon flow. We executed the scan at a rate of 1 Hz with a resolution of 512 × 512 pixels. We performed sample analysis using the software “Analysis Section” of the equipment system. The values represent the mean ± SD derived from approximately 40 particle measurements. 

#### 2.3.3. Determination of Artemether Encapsulation

We determined the ATM content using an HPLC-validated method as described [21,22,36]. The system consisted of HPLC (Waters Alliance 2695, Waters Inc., Milford, MA USA) coupled to a UV detector at a wavelength of 216 nm. We used an RP-C18 Gemini Phenomenex column (150 mm × 4.6 mm) column with 0.5 μm of particle size, protected by a security guard column AJ0-4287 C18 (0.5 mm × 4.6 mm, 0.5 μm) (Phenomenex Inc., Torrance, CA, USA) at 30 °C. The mobile phase was acetonitrile/water (70:30 *v/v*) pumped at a flow rate of 1 mL/min (isocratic) and 50 µL of samples were injected, with a run time of 8 min each. The retention time was 6.35 min. We determined the total (100%) of ATM present in NCs suspension by diluting 400 μL of its in acetonitrile/ethanol (1:1). We assessed free ATM soluble in the aqueous external phase by ultrafiltration/centrifugation method in AMICON units (Microcon^®^, MWCO 100,000, Millipore^®^) of 400 μL suspension at 500 × *g* for 30 min. We calculated the ATM encapsulation yield (drug loading %) in nanostructures as the difference between the total amount of ATM in the final colloidal suspension after filtration through a 0.8-μm (Millex^®^-Millipore) membrane; and the amount of free soluble drug in the external aqueous phase of the colloidal suspension, divided by the total amount of ATM in the NCs dispersion. The encapsulation efficiency (EE%) takes into account the losses of the ATM in the total process, and it is calculated as a percentage of the amount of ATM truly encapsulated (400 μL) in suspension divided by the total of drug added to produce the formulation (400 μL) [37] using the equations below:

Drug loading % = total ATM in final NC dispersion − ATM in ultrafiltrate × 100

total ATM in the final NC dispersion

Encapsulation efficiency % = total ATM in the final NC dispersion − ATM in the ultrafiltrate × 100

total drug feed in the formulation

#### 2.3.4. In Vitro Artemether Release from Nanocapsules

We determined the ATM solubility in water and PBS pH 7.4 using the HPLC method described above. The ATM thermodynamic solubility was 22.42 µg/mL in water and 14.54 µg/mL in PBS. In vitro release studies and assessment of ATM dissolution rate from NCs were conducted using the inverted dialysis method [38] in phosphate-buffered saline (PBS pH 7.4) under sink conditions (20% of saturation solubility) previously determined as 14 µg/mL at 37 °C [36]. Free-form ATM crystals (1.4 mg) or 350 µL of ATM-PCL-NCs (concentration of 4 mg/mL) were placed at time 0 in 500 mL of PBS release media at 37 °C in a thermostatic shaker bath containing seven dialysis sacks (Spectra Por 12,000–14,000 Da MWCO) with 1 mL de PBS. At each time interval (0, 5, 30, 60, 120, 360, 720, 1440 min), a dialysis sack was withdrawn simultaneously with a sample of release media (500 µL). The samples were diluted 1:1 with acetonitrile, then vortex mixed, centrifuged at 500× *g* for 5 min, and supernatant assayed by HPLC-UV to determine ATM concentrations using a validated method [21,36]. Three independent experiments were conducted; with each one tested in triplicate.

### 2.4. Experimental Animals

All procedures related to animal use conformed with the Ethical Principles of Animal Experimentation (Brazilian College of Animal Experimentation) and were approved by the UFOP Ethics Committee under number 03/2011. 

#### Antimalarial efficacy in *Plasmodium berghei*-Infected Mice

Mice represent an ideal rodent model for *Plasmodium berghei* infection, which is fatal for these animals [39]. This experimental model simulates the human infection produced by *Plasmodium falciparum*, which is also lethal in non-treated humans. We used two treatment protocols to determine ATM efficacy in the mouse model. The first one was applied early after mice infection with high doses (total 40 mg and 80 mg/kg divided into 4 daily doses) following the “Four-day test” protocol [13,39]. This protocol aims to evidence efficacy and any general toxicity of the formulations in infected animals in repeated dose experiments. Efficacy was evaluated against chloroquine-sensitive *P. berghei* NK65-infected mice.

The second efficacy protocol consisted of the treatment of infected mice with established infection (15% blood parasitemia) with a single low dose of ATM (20 mg/kg IV) to better distinguish the formulation profile of the efficacy following time. An infective inoculum was prepared from an infected donor mouse with rising parasitemia (20%). Swiss female mice (18–22 g) were infected (IV) on day zero with 1 × 10^6^ *P. berghei* parasitized red blood cells (RBC) in 0.2 mL of phosphate-buffered saline and randomly divided into groups. Then, the animals were treated on day 2, with only one IV dose of 20 mg/kg free-ATM solution or ATM-PCL-NCs. Control groups received only ATM vehicles or blank PCL NCs (blank-NCs). Thin blood smears were prepared with blood collected from the tail vein of all animals on days 3, 5, 7, 9, 14, 25, and 60 after infection. Parasitemia was measured in Giemsa-stained smears, counting at least 3000 RBC to determine the percentage of infected ones.

### 2.5. Determination of Cardiovascular Parameters and Protocols

**Figure 1:**
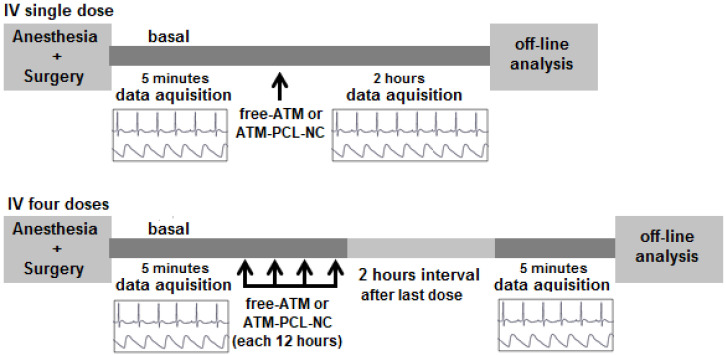
Experimental protocol of vehicle, blank-NC, free-ATM, and ATM-PCL-NC treatment for ECG and AP signal register.

The rat is the rodent model of choice to study cardiotoxicity using electrocardiogram (ECG) analysis and for measurements of arterial blood pressure. We used male Wistar rats (200–220 g). We anesthetized the animals with ketamine (100 mg/kg) and xylazine (14 mg/kg) mixture by intraperitoneal route. The femoral artery and vein were catheterized under anesthesia to allow recording of the AP and IV drug administration, respectively. AP was recorded using a disposable pressure transducer (TruWave, Edwards Life Sciences, Irvine, CA, USA) connected to a signal conditioning system. Limb lead II of ECG was continuously recorded using subcutaneous stainless steel needle electrodes connected by a shielded cable to a biopotential amplifier, with all the care related to the frequency response and the characteristics of the recorded signals [21]. The output signals of these systems were sampled at 1200 Hz by a 16-bit A/D conversion board (DaqBoard/2000, IOtech, Cleveland, OH, USA) and stored on a PC hard disk. We used two protocols to evaluate the cardiovascular safety of ATM in solution (free-ATM) or loaded in NCs (ATM-PCL-NCs) (Figure 1).

Single IV dose: We recorded the ECG and AP signals continuously for 5 min before and after a single IV injection of the different formulations at 40 or 80 mg/kg. Thereafter, segmented data records of 30 s were performed every 5 min up to 30 min and every 15 min up to 2 h after the administration of the different formulations.

Four IV doses: The ECG and AP signals were recorded continuously for 5 min as basal controls of each animal. Then, the animals received four IV doses (ATM 20 mg/kg) of the different formulations every 12 h for 48 h. Thereafter, 2 h after the last injection, signal records of 5 min were obtained.

From the stored records analyzed offline, two seconds of segments (raw data) containing 6–12 heartbeats depending on the heart rate (HR) were extracted, and all the cardiovascular parameters were calculated as a mean value of these segments (filtered data). The cardiac parameters extracted from ECG records were the QT interval (interval between the beginning of the Q-wave and the end of the T-wave), RR interval (interval between two successive R-waves and used to obtain the HR = 60/RR), PR interval (interval between the beginning of the P-wave and the end of the R-wave), and QRS (interval from the beginning of the Q- wave to the end of the S-wave) interval. The QT interval was corrected to obtain QTc, using Fridericia’s formula (QTc = QT/(RR)^1/3^), to correct its HR dependence [22]. The cardiovascular parameters extracted from AP signals were systolic (SAP) and diastolic blood pressure (DAP). We calculated the percentual variation using the parameter value of each point related to the basal period, taken as a control of the same animal. 

### 2.6. Statistical Analysis

We used the Kolmogorov–Smirnov method to determine whether continuous variables were normally distributed. We expressed the in vivo results as mean ± S.E.M. We performed statistical comparisons using ANOVA and Tukey’s post-test. We accepted significance when *p* < 0.05.

## 3. Results

### 3.1. Nanocapsules Characterization and ATM Release Rate

Table 1 shows the physicochemical characterization of ATM NCs. The ATM encapsulation yield (drug loading %) was close to 100% for all types of NCs using the different polyesters. Blank-NCs for all formulations had an average size below 250 nm. However, after ATM association, the system increased in size and polydispersity. These larger sizes are related to the association of ATM with the oily core of the NCs. Loading of ATM in PLA-NCs increased the average particle size and polydispersity with increasing concentrations of ATM compared to the other polyester NC. On storage, PEG-PLA and PLA NCs showed a more unstable profile, and aggregations were observed seven days after preparation. Sizes larger than 300 nm are not suitable for IV administration.

**Figure 2:**
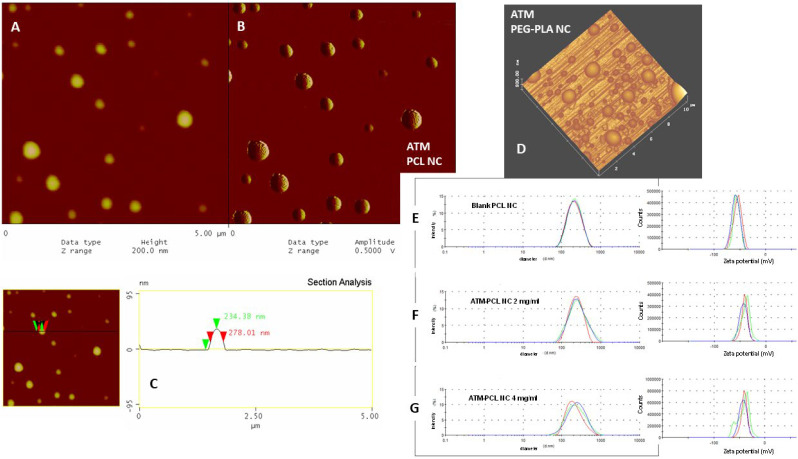
Morphological characterization of nanocapsules by atomic force microscopy (AFM) in (**A**–**D**) images, zeta potential, and size distribution by intensity determined by dynamic light scattering (DLS) in (**E**–**G**) graphs. (**A**) is the AFM height and (**B**) is the corresponding AFM phase images of ATM-PCL-NC 2 mg/mL and (**D**) is the 3D AFM image of ATM-PEG-PLA NC 2 mg/mL showing the size dispersion of spherical particles deposited under mica plates. Scheme (**C**) is the topographical profile of a selected NC in image (**A**) showing the measurement of diameter at half-height and the values measured by the equipment. In (**E**): blank-PCL-NC; (**F**): ATM-PCL-NC 2 mg/mL and (**G**): ATM-PCL-NC 4 mg/mL formulations.

The best formulation in terms of average size was ATM-PCL-NCs (Table 1 and Figure 2). The polydispersity index was less than 0.3 consistent with the size of monodispersed populations for PCL loaded with ATM at concentrations of 2 and 4 mg/mL. The mean size increased (by 35–45 nm) after loading with ATM. The association with ATM influenced the zeta potential in different ways depending on the polyester used in the formulation. However, PLA and PEG-PLA produced less stable particles with the highest polydispersity. Thus, the zeta potential was also affected by the heterogeneity of the system. In general, ATM influences the zeta potential, and it seems that part of the drug is localized on the surface of NCs. The PCL-NCs loaded with ATMs were the most stable in their physicochemical profile. They were selected for biological evaluation. Examination of the ATM-PCL-NCs morphology by AFM height and phase images showed monodispersed populations of particles. In contrast, AFM analysis showed that ATM-loaded PEG-PLA NCs were more polydispersed (Figure 2). The mean diameter extracted from the AFM analysis was greater than that measured by DLS, and the diameter/height ratio was greater than one in the topographical profile (Figure 2). This aspect is in agreement with the ability of NCs to deform under the pressure of the AFM tip. We also highlighted the higher heterogeneity of PEG-PLA NC in size dispersion by 3D AFM images of NCs in Figure 2D.

**Figure 3:**
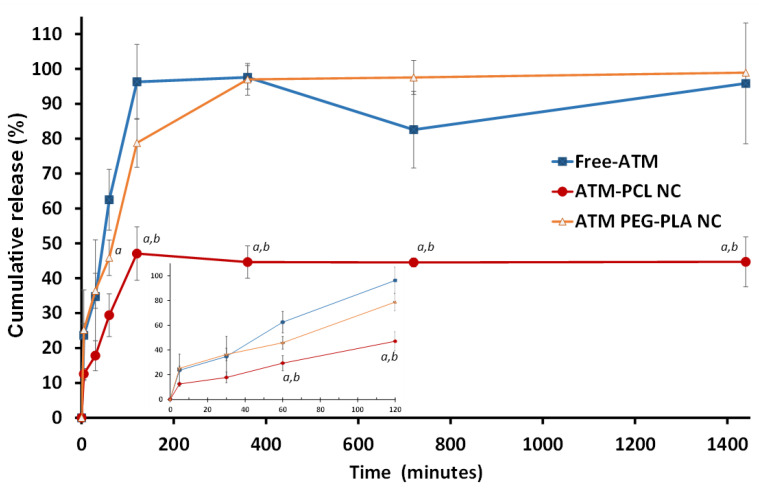
Profiles of the cumulative release of free-artemether (Free-ATM) and artemether from nanocapsules of polycaprolactone and PEG-PLA polymers following time in PBS pH 7.4 under *sink* conditions in 37 °C using inverted dialysis sac method. The insert represents the first 2 h. Data shown are means ± standard deviations of n = 3 independent experiments. The assay was made in triplicate (nine measurements in total). *^a^* is a mean statistically different from free-ATM and *^b^* statistically different from ATM-PEG-PLA NC. The values at each time point were compared using an unpaired student’s *t*-test.

Figure 3 shows the ATM dissolution profile in PBS 7.4 and the release profile of the ATM from PCL-NCs and PEG-PLA NCs determined by the reverse dialyze membrane method. The PEG-PLA showed a fast release of ATM (25%) in the first 5 min immediately after dilution in release media followed by a complete release within the next 6 h. This profile was similar to the free-ATM dissolution in this medium under *sink* conditions (20% of ATM saturation solubility). In contrast, PCL-NCs showed a burst release of approximately 12% in the first minutes followed by a release of 50% of total ATM encapsulated for up to 2 h. The 50% amount was not released and was maintained associated with the nanocarrier even under *sink* conditions. We considered the profile of the ATM loaded in PCL-NCs to be the most suitable for the biological assessment of infection and cardiovascular toxicity models in vivo.

### 3.2. Antimalarial Efficacy in P. berghei-Infected Mice

**Figure 4:**
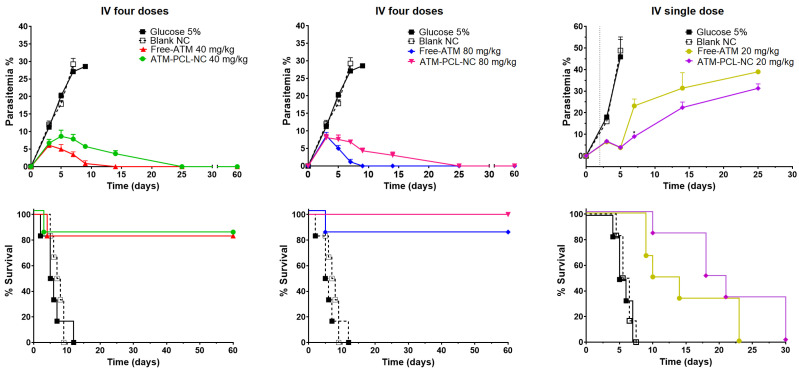
Efficacy of the artemether formulations represented in graphs of parasitemia (%) and survival (%) for both protocols (four-day-test) and single low dose (20 mg/kg) with established parasitemia (15%).

The antimalarial efficacies of ATM-PCL-NCs and free-ATM were similar in Swiss mice infected with *P. berghei*. We treated mice with low parasitemia using 40 or 80 mg/kg divided into four daily doses delivered by the IV route. These are high doses and no systemic toxicity was observed in the infected mice. An untreated group was evaluated concurrently for each protocol. Control groups (IV solution vehicle, and blank-NCs) remained highly parasitized. All the animals died between day 5 to day 10 (Figure 4). The IV treatment with free ATM or ATM-PCL-NCs at 40 or 80 mg/kg/day for four consecutive days reduced parasitemia to very low levels, avoiding the progression of the infection, and increased mice survival (Figure 4). The success of the cure was verified in all groups treated with ATM for up to 10 days. No recrudescence occurred 60 days after treatment. Of note, there was no difference in survival profile and parasitemia levels in animals treated with blank NCs and glucose solution, indicating no antimalarial effect of blank-PCL-NCs. There was no difference between the two doses and formulations concerning parasitemia and survival after repeated doses (Figure 4).

In the second treatment efficacy protocol (Figure 4-IV single dose), the aim was to distinguish the effect of the formulation with a low single dose of ATM in mice with established infection (parasitemia of 15%). ATM-PCL-NCs improved mice survival and reduced parasitemia more rapidly than single doses of the free-ATM solution (Figure 4).

### 3.3. Determination of Cardiovascular Effects of ATM

**Figure 5:**
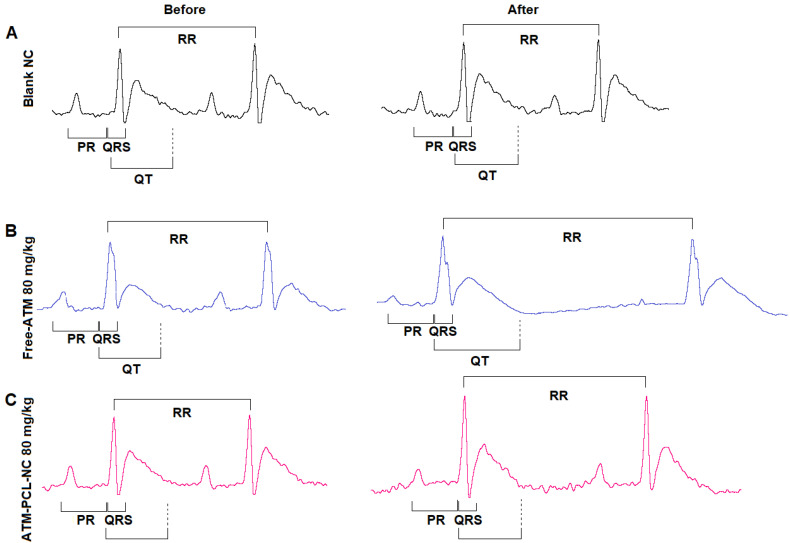
Representative ECG signal (lead II) at basal (before treatment) and after treatment with Blank-NC in (**A**), Free-ATM in (**B**) and ATM-PCL-NCs in (**C**), showing the ECG intervals analyzed. The RR interval was used to obtain the heart rate (HR).

**Figure 6:**
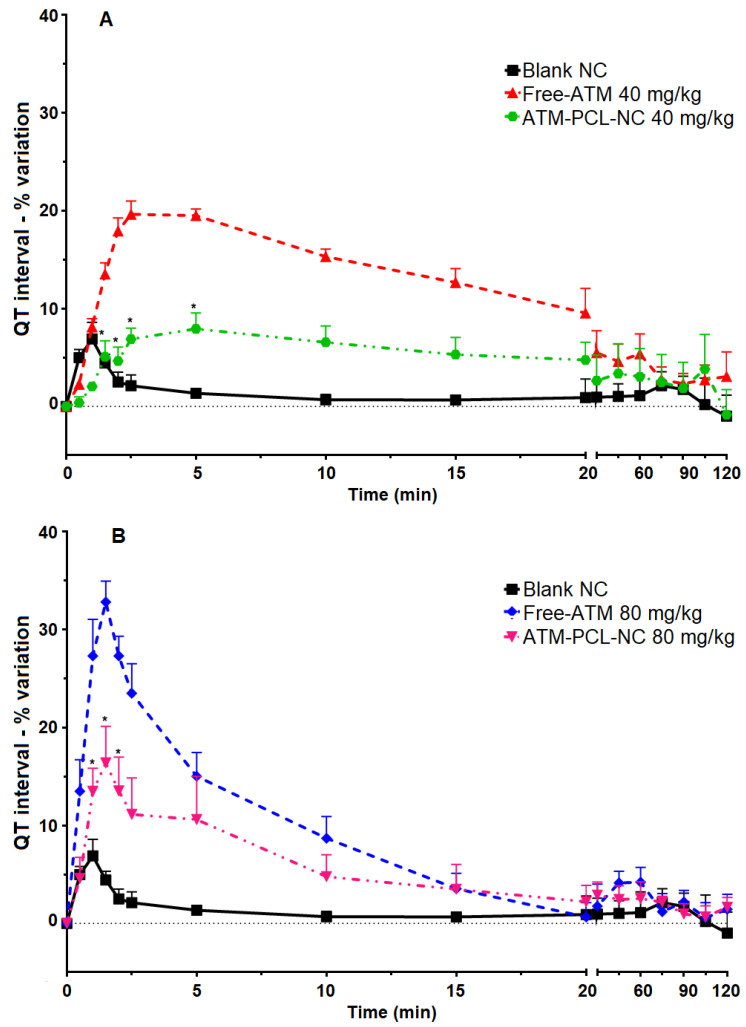
QT interval percentual variation from basal (before treatment) until two hours after IV single dose administration of blank-NC, free-ATM, and ATM-PCL-NC, both at 40 (**A**) and 80 mg/kg (**B**). * *p* > 0.05 compared to free-ATM administration. ANOVA followed by Tukey post-test.

Figure 5 shows representative ECG signals before and after IV administration of blank NCs, free-ATM, and ATM-PCL-NCs in rats. IV administration of free-ATM increased the QT and QTc intervals of the ECG, occurring rapidly (between one to twenty minutes after delivery), which persisted until the end of the experiment (Figure 6). The effects were dose-dependent. Administration of ATM-PCL-NCs induced less prolongation of the QT and QTc intervals. In contrast, free-ATM or ATM-PCL-NCs did not affect the PR and QRS intervals of the ECG relative to the basal period. Additionally, we investigated free-ATM at 120 mg/kg in pilot experiments but, at this dose, the majority of animals died during the experiment with ECG alterations and arrhythmia (data not shown).

**Figure 7:**
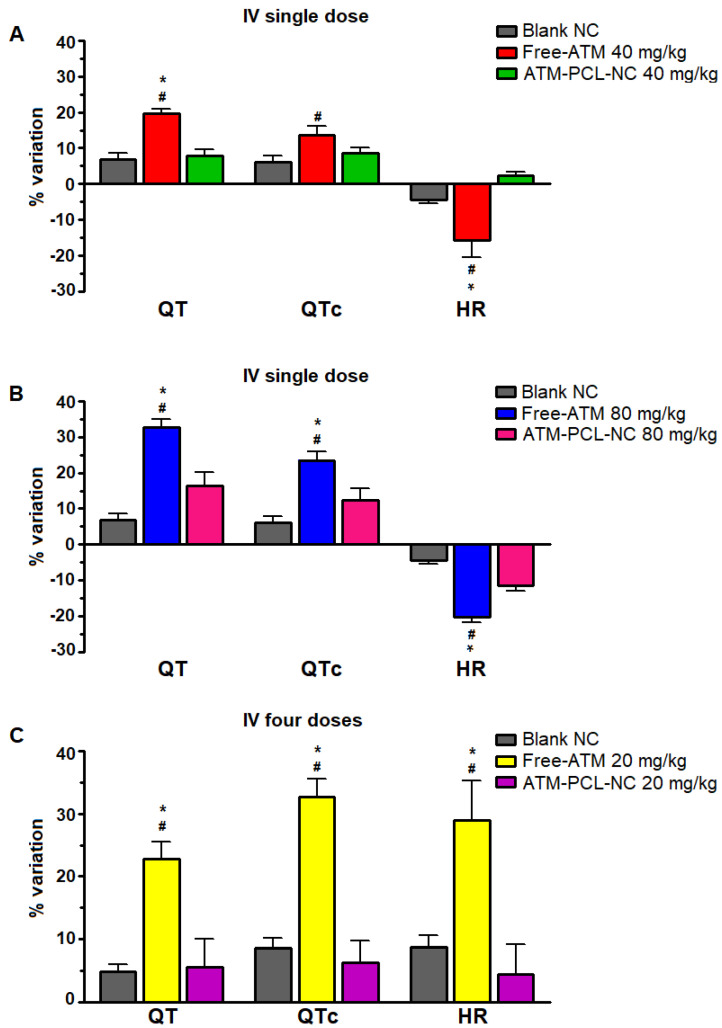
Maximal percentual variation of QT and QTc intervals and heart rate (HR) after IV administration of blank-NC, free-ATM, and ATM-PCL-NC, a single dose of 40 (**A**) and 80 mg/kg (**B**), and four doses (one every 12 h) of 20 mg/kg (**C**). * *p* > 0.05 compared to blank-NC and # *p* > 0.05 compared to ATM-PCL-NC administration. ANOVA followed by Tukey post-test.

**Figure 8:**
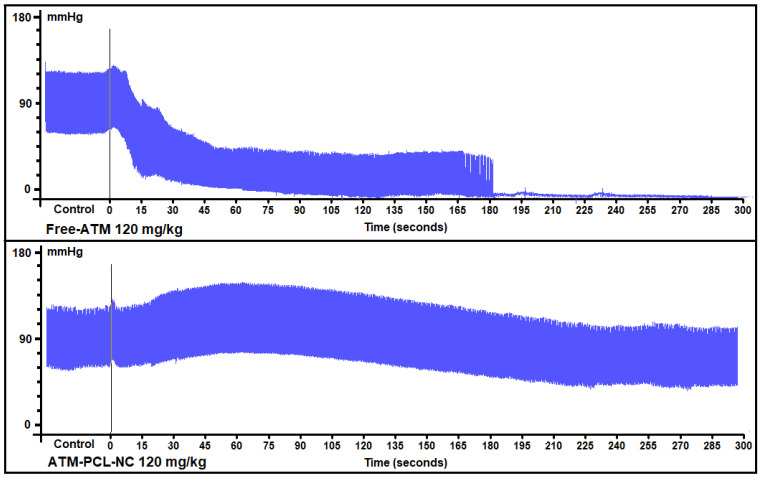
Representative signals of arterial blood pressure of anesthetized rats treated with free-ATM or ATM-PCL-NC, both at 120 mg/kg, showing the severe hypotension produced by the free form and the absence of hypotension with the NC formulation.

**Figure 9:**
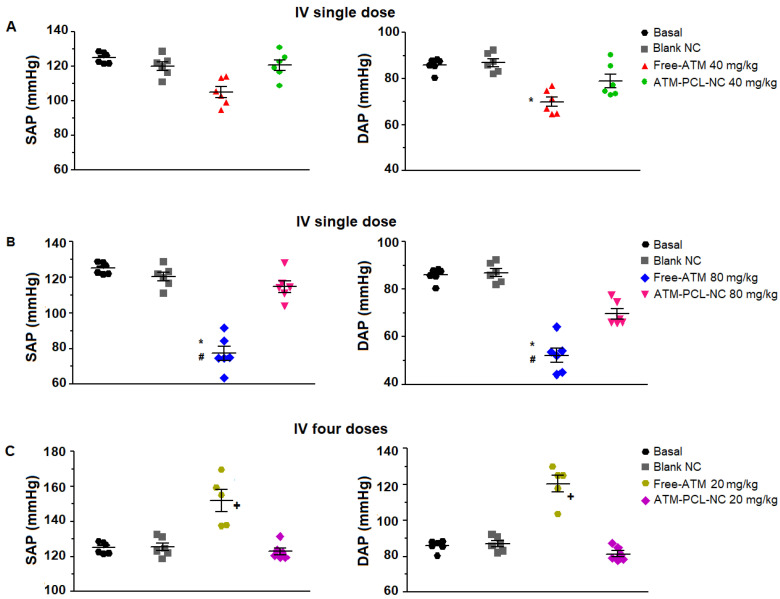
Maximal variation of systolic (SAP) and diastolic (DAP) pressure (mmHg) after IV administration of blank-NC, free-ATM, and ATM-PCL-NC, a single dose of 40 (**A**) and 80 mg/kg (**B**), and four doses (one every 12 h) of 20 mg/kg (**C**). * *p* < 0.05 compared to blank-NC and # *p* < 0.05 compared to ATM-PCL-NC administration, ^+^ *p* < 0.05 compared to basal, blank-NC, and ATM-PCL-NC. ANOVA followed by Tukey post-test.

Figure 7 shows the maximum changes in cardiovascular parameters measured before and after ATM administration of a single dose of 40 mg/kg (A) or 80 mg/kg (B), and of the four doses (20 mg/kg) in 48 h (C). After the single IV dose, the QT interval increased by 19.6% and 32.8%, respectively for 40 mg/kg and 80 mg/kg of free ATM. Encapsulation of ATM in NCs significantly prevented ATM-induced QT interval prolongation (*p* < 0.05), with approximately 60% and 50% of reduction after IV administration of 40 mg/kg or 80 mg/kg, respectively. Similarly, for the group receiving four repeated doses of 20 mg/kg free-ATM, the QT interval increased from 66.5 ms (basal) to 81.6 ms (two hours after the last dose of ATM-PCL-NCs). After the single dose, free-ATM reduced the blood pressure (SAP and DAP) (Figure 8 and Figure 9A,B), and HR (Figure 7A,B), mainly from one to 20 min post-administration (Figure 8 and Figure 9). The hypotension was severe with free-ATM (Figure 8). The decreases were, respectively for free-ATM doses of 40 mg/kg and 80 mg/kg, 17.8% and 41.4% for SAP, and 22.2 % and 49.9% for DAP. We observed no effect after the administration of the control solution or blank-NCs. The effects were weaker with the administration of ATM-PCL-NCs inducing a reduction of 9.8% and 13.8% for SAP and 8.5% and 16.4% for DAP for a single dose of 40 mg/kg or 80 mg/kg, respectively (Figure 9A and B). On the other hand, after the four doses protocol, free-ATM increased the HR (+76 bpm; Figure 7C), SAP (+26.1%), and DAP (+37.8%) (Figure 9C). The final absolute values of AP and HR in this group indicate substantial hypertension and tachycardia. These effects were reduced when ATM-PCL-NCs were administered, being only +4.4% for HR, and +3.6% and +5.1% for SAP and DAP, respectively. In summary, all cardiovascular alterations indicating cardiovascular toxicity of free-ATM were markedly reduced when the drug was administered as ATM-PCL-NCs (IV route). Encapsulation in our NCs formulation prevented alterations of the ECG parameters and blood pressure of ATM.

## 4. Discussion

In this work, we sought to overcome the main limitations of ATM treatment by developing new formulations designed for intravenous administration for different therapeutical purposes. For example, to treat severe malaria successfully, rapid suppression of parasitemia using IV ATM administration as well as a sustained ATM release into the blood is highly desirable. In addition to the treatment of malaria, these formulations could also be useful for other uses of ATM in therapeutic repositioning strategies against schistosomiasis and cancer [6,7]. Difficulties concerning current treatment regimens include low water solubility of ATM, short half-life, fast degradation, and adverse effects. ATM is a lipophilic substance and, as such, NCs, with their oily core reservoir, make an ideal nanocarrier that can protect the ATM from degradation [26] and allow a controlled release after IV injection [19].

In this study, we demonstrate the development of NCs, prepared from different biodegradable polyesters, as intravenous formulations of ATM to treat severe malaria. These polymers are commercially available in high-purity grades with low dispersity and are approved for IV administration [24,25,40]. They can control the release of drugs into the blood as previously reported [19,22]. We prepared the NCs using a solvent displacement technique [34]. Advantages are the narrow distribution of NCs sizes and the use of well-defined and safe biodegradable polymers, such as polyesters, non-toxic excipients, and easily removable non-chlorinated solvents [24,25]. ATM has been encapsulated in PCL-NCs at high payloads, as much as 2 or 4 mg/mL of ATM, with an encapsulation efficiency of 92% and 80% respectively, in only 6 mg/mL polymer with a low drug interference at NC surface. This drug loading is promising compared with other lipophilic substances loaded into lipid nanocarriers [27,28,29,30,41,42,43]. For example, the optimized formulation of ATM in nanoemulsion was 0.4 mg/mL [42] and ATM showed 65% of encapsulation efficiency in nanoliposomes [44]. ATM in nanostructured lipid carriers with 87% of ATM encapsulation efficiency and 11% payload *w*/*w* were reported [41]. We found similar yields with ATM payload of 13% *w*/*w* in NC. Our data indicates a high affinity of the ATM for the oily core of PCL-NCs. The mean hydrodynamic diameters and the polydispersity indexes of both concentrations of ATM-PCL-NCs (2 and 4 mg/mL) are suitable for IV administration (Table 1). Particle size distribution must be strictly controlled in nanoparticulate formulations intended for intravascular delivery to prevent blood capillary occlusion [44]. ATM has already been associated with different types of lipid nanocarriers to be used in different routes of administration [27,28,29,30,41,42,44]. However, in these studies, no assessment of efficacy or cardiotoxicity upon intravenous administration was provided.

The morphology of polyester NCs prepared from PCL, PLA, and PEG-PLA polymers was characterized using different techniques as previously reported [19,20,26,45,46,47]. TEM and SEM techniques are useful, yet the shrinking and melting of these polymeric systems are frequently observed under electron beam incidence [18] as well as artifacts related to staining and coating [48]. The AFM technique in *tapping* mode has been employed recently to obtain information regarding the morphology [48,49], particle dispersion, stiffness, deformation [45,46], encapsulation [50], tip interaction with the surface [51], and surface porosity of nanospheres [52] and nanocapsules [18,45,46]. For these types of polymer nanocapsules, AFM provides much more additional physical information than electron microscopy. The AFM image analysis we performed indicates that NC, in contrast to hard nanospheres, can deform under tip pressure. We provide evidence in the AFM section analysis that the flattening of the ATM-PCL-NCs occurs under mica plates, based on the topographical profile of these NCs showing a diameter/height ratio higher than one (Figure 2C). This deformable property of the NCs structure, with an oily core surrounded by a polymeric wall-forming reservoir, is suitable for IV administration [44].

The negative value of PCL-NC zeta potential was influenced by ATM, indicating that part of the ATM was associated with the surface of the carrier. The presence of ATM at the NCs surface was confirmed by the ATM *burst* type release in PBS media in the first 5 min. However, as the NCs’ surface charges are all very negative (>30 mV), we observed no impact on colloidal stability. The ATM-PCL-NC formulations were physically stable during the experimental period with no signs of aggregation, flocculation, or phase separation.

The hydrophobicity of the polyester also plays a role because PEG-PLA and PLA polymers with lower hydrophobicity were less efficient at encapsulating ATM in the nanocarrier. This behavior affects the release of ATM. PCL controls the release rate of ATM better than PEG-PLA under sink conditions, in line with the ability of this polymer to modify the release rate into blood (Figure 3). The size, polydispersity, and release profile of PCL NCs showed that this formulation was suitable for IV administration with a modified biodistribution allowing for sustained release of ATM and reduced toxicity.

The efficacy testing was conducted in a rodent model of malaria consisting of mice infected with *Plasmodium berghei,* which is one of the best models to simulate the fatal *Plasmodium falciparum* infection in humans. *Plasmodium berghei* produces an asynchronous infection in mice with high parasitemia that results in mortality if left untreated. We employed two protocols, one with low parasitemia and the other with high and established parasitemia. Importantly, the four-day test confirmed the anti-malarial efficacy of ATM-containing NC formulations in *P. berghei*-infected Swiss mice with no detectable adverse effects after four repeated doses. The IV administration of free and encapsulated ATM at total doses of 40 mg/kg and 80 mg/kg induced complete remission of parasitemia, with no recurrence of the infection for up to 60 days. Such efficacy has been reported for ATM combined with solid lipid nanoparticles [29], or when administered in NCs orally [22]. In a more stringent protocol at a single low dose (20 mg/kg) and high parasitemia (15%), ATM-PCL-NCs showed a faster reduction of parasitemia and a more sustained effect than the free-ATM solution delivered by IV injection.

Given the effectiveness of ATM in an encapsulated form on parasitemia of *P. berghei*-infected mice, we raised the legitimate question of whether, and to what extent, this property could induce cardiovascular toxicity. The determination of cardiotoxicity using QT interval prolongation is currently used, and mandatory, for decision-making during drug development [53,54]. Different antimalarial drugs are associated with decreases in both HR and AP as a cause of sinus bradycardia and fatal hypotension [55,56]. ECG recordings have shown that QT interval prolongation, a strong marker of potential cardiotoxicity [53], is associated with potentially lethal tachycardia known as ‘*torsade de pointes*’ degenerating into ventricular fibrillation [55,56]. In our experiments, the two protocols used (either a single dose of 40 mg/kg or 80 mg/kg; or repeated administration of 20 mg/kg) prolonged the QT and the QTc interval.

The effects of ATM on HR and AP were more complex and rather counterintuitive. The two single doses (40 and 80 mg/kg) rapidly induced bradycardia and severe hypotension (decrease of SAP and DAP). In contrast, repeated administration of four doses of ATM at 20 mg/kg induced tachycardia and hypertension. Why this occurs is unclear. However, one possibility is that the treatment evokes a time-dependent impact on the sympathovagal balance with an immediate enhancing parasympathetic effect accounting for the bradycardia and hypotension. In this scheme, repeated episodes of ATM injection may activate a regulatory feedback mechanism to counteract vagal predominance and boost the sympathetic contribution, as indicated by tachycardia and hypertension. Although ATM has direct effects on the cardiomyocyte action potential, in line with the lengthening of the QTc interval, and abnormal Ca^2+^ handling [21], toxicity on the central nervous system has also been suggested [31]. Moreover, the targeting of membrane-delimited proteins involved in the electrical activity of neurons or vascular cells may also account for these observations.

A key outcome of our study was evidence of a safer cardiovascular profile of ATM at effective antiparasitic doses on *P. berghei* when ATM was released from the engineered NCs. Encapsulation of ATM in NCs reduced all adverse effects induced by either the two single doses (40 mg/kg and 80 mg/kg) or the four repeated doses (20 mg/kg) of ATM administered intravenously. This point is a novel finding concerning ATM encapsulated in nanocarriers. The modifications of cardiac parameters, such as the HR, QT, and QTc intervals, as well as blood pressure, were largely attenuated. Of note, while the adverse effects of ATM on HR and AP were the opposite, depending on the administration protocol, NCs were efficient in preventing or minimizing them. Formulation design and administration route play a key role in drug plasma concentration. Although it appears that there is a discrepancy between the antiparasitic efficacy of ATM and the attenuation of its cardiovascular side effects provided by PCL-NCs, the differences may be inherent to the cell types (proliferative organisms versus differentiated mammalian cells). In particular, the activity against *P. berghei* possibly relates to the continuous blood release of an efficient antiparasitic drug sustaining the antiparasitic effect. The lower blood ATM dose, therefore, may have less systemic toxicity, particularly in the cardiovascular system. A fraction of free ATM, smaller than that observed following IV delivery of a high concentration of the free-ATM, may thus reach the cardiac tissue. Indeed, the toxicity of artemisinin derivatives has been correlated with drug concentration in the blood [10]. A modification of ATM biodistribution due to NC accumulation in the mononuclear phagocytic system after IV administration may also occur [57].

## 5. Conclusions

Our data show that the ATM-PCL-NCs maintain ATM antimalarial efficacy but have less associated cardiotoxicity than the free-ATM solution upon IV administration. The ATM-PCL-NCs exhibited the most favorable profile of ATM release compared to other engineered biodegradable polymers in our study. Furthermore, NC has an oily core where oil-soluble substances can be dissolved, resulting in high payloads. In this sense, the association of the ATM with NCs is a promising strategy for ATM IV administration. The modification of drug distribution using polymeric NCs appears as a strategy to be applied to other lipophilic, pro-arrhythmogenic drugs. This study also contributes to an important in vivo methodology to measure the cardiotoxicity of nanoparticulate drug carriers. In summary, the use of polymeric NCs represents a promising strategy for the production of safer IV treatments with a large plethora of cardiotoxic drugs.

## Figures and Tables

**Table 1:** Characterization of the artemether-loaded nanocapsules.

Polymer/NCs	ATM mg/mL	Hydrodynamic Diameter (nm) ^a^	Polydispersion Index ^b^	Zeta Potential (mV)	Encapsulation Yield or Drug Loading (%)	Encapsulation Efficiency (%) ^#^
Blank PCL	0	197.3 ± 0.8	0.138 ± 0.01	−56.2 ± 1.7	-	-
ATM-PCL 2	2	232.1 ± 2.7 *	0.266 ± 0.03	−49.3 ± 1.6 *	98.52 ± 0.3	91.81 ± 0.6
ATM-PCL 4	4	243.2 ± 4.7 *	0.278 ± 0.04	−41.9 ± 1.3 *	93.91 ± 0.5	80.03 ± 0.7
Blank PLA	0	256.7 ± 0.9	0.245 ± 0.05	−46.6 ± 1.3	-	-
ATM-PLA	0.5	251.9 ± 0.9	0.208 ± 0.07	−40.8 ± 2.1	ND	ND
ATM-PLA	1	301.5 ± 6.5 *	0.32 ± 0.15 *	−56.2 ± 0.6 *	90.22 ± 0.4	85.81 ± 0.8
ATM-PLA	2	328.7 ± 5.7 *	0.378 ± 0.41 *	−51.9 ± 2.3 *	87.11 ± 0.5	73.3 ± 0.7
Blank PEG-PLA	0	222.7 ± 3.4	0.17 ± 0,05	−54.4 ± 3.5	-	-
ATM PEG-PLA	1	296.6 ± 1.5 *	0.22 ± 0.05	−61.2 ± 1.2 *	94.5 ± 0.5	71.0 ± 2.8
ATM PEG-PLA	2	343.5 ± 4.7 *	0.58 ± 0.08 *	−63.8 ± 4.1 *	87.9 ± 1.2	74.8 ± 0.7

Values represent the mean ± standard deviation derived from 3 preparations; Refers to the freshly prepared formulation; ^#^ Refers to the total drug added to prepare the formulation (losses in the process). ^a^ Measured by dynamic light scattering. ^b^ Mean polydispersity index, (<0.3) monodispersed samples; The sizes and zeta potential values were compared using unpaired student’s *t*-test. * Significantly different from the respective blank-nanocarrier (*p* < 0.05). ATM is artemether. PCL is polycaprolactone, PLA is homopolymer polylactide, and PEG-PLA is a *diblock* amphiphile polymer. ND: not determined.

## Data Availability

The data presented in this study are available on request from the corresponding author.
